# One-year mortality after severe COPD exacerbation in Bulgaria

**DOI:** 10.7717/peerj.2788

**Published:** 2016-12-13

**Authors:** Evgeni Mekov, Yanina Slavova, Adelina Tsakova, Marianka P. Genova, Dimitar T. Kostadinov, Delcho Minchev, Dora Marinova, Mihail A. Boyanov

**Affiliations:** 1 Clinical Center for Pulmonary Diseases, Medical University—Sofia, Sofia, Bulgaria; 2 Department of Clinical Laboratory and Clinical Immunology, Central Clinical Laboratory, Medical University—Sofia, University Hospital “Alexandrovska,”Sofia, Bulgaria; 3 Department of Internal Medicine, Clinic of Endocrinology and Metabolism, Medical University—Sofia, University Hospital “Alexandrovska,”Sofia, Bulgaria

**Keywords:** COPD, Exacerbation, Mortality, Lung function, Quality of life, Comorbidities

## Abstract

**Introduction:**

One-year mortality in COPD patients is reported to be between 4% and 43%, depending on the group examined.

**Aim:**

To examine the one-year mortality in COPD patients after severe exacerbation and the correlation between mortality and patients’ characteristics and comorbidities.

**Methods:**

A total of 152 COPD patients hospitalized for severe exacerbation were assessed for vitamin D status, diabetes mellitus (DM), arterial hypertension (AH), and metabolic syndrome (MS). Data were gathered about smoking status and number of exacerbations in previous year. CAT and mMRC questionnaires were completed by all patients. Pre- and post-bronchodilatory spirometry was performed. One-year mortality was established from national death register.

**Results:**

One-year mortality is 7.2%. DM, MS, and VD are not predictors for one-year mortality. However there is a trend for increased mortality in patients with AH (9.5% vs. 2.1%, *p* = 0.107). There is increased mortality in patients with mMRC > 2 (11.1 vs. 0%, *p* = 0.013). The presence of severe exacerbation in the previous year is a risk factor for mortality (12.5% vs. 1.4%, *p* = 0.009). There is a trend for increased mortality in the group with FEV1 < 50% (11.5 vs. 4.4%, *p* = 0.094). Cox regression shows 3.7% increase in mortality rate for 1% decrease in FEV1, 5.2% for 1% decrease in PEF, 7.8% for one year age increase and 8.1% for 1 CAT point increase (all *p* < 0.05).

**Conclusions:**

This study finds relatively low one-year mortality in COPD patients after surviving severe exacerbation. Grade C and FEV1 > 80% may be factors for good prognosis. Risk factors for increased mortality are age, FEV1 value, severe exacerbation in previous year and reduced quality of life.

## Introduction

Chronic obstructive pulmonary disease (COPD) is a preventable and treatable disease which is associated with significant extrapulmonary effects. By 2030, COPD is expected to be the fourth highest cause of mortality worldwide. The prognosis of COPD patients could be influenced by the extrapulmonary comorbidities (GOLD, 2016).

One-year mortality in COPD patients is 4.3–43%, depending on the subgroup examined (Table 1) (Connors et al., 1996; Almagro et al., 2002; Groenewegen, Schols & Wouters, 2003; Kim, Clark & Camargo, 2006; Coleta et al., 2008; Fan et al., 2007; Gershon et al., 2010; Piquet et al., 2013; Ho et al., 2014; Müllerova et al., 2015). Mortality shows a downward trend in recent studies. Identified risk factors for increased mortality are: severe exacerbations, reduced FEV1, age, reduced quality of life, hypoxemia, and presence of comorbidities. COPD patients with severe exacerbation have increased mortality rate. Among comorbidities, vitamin D deficiency and insufficiency, diabetes mellitus (DM), and metabolic syndrome (MS) are associated with increased mortality (Divo et al., 2012; Gudmundsson et al., 2006; Galassi, Reynolds & He, 2006; Mannino et al., 2008), but not in all studies (Benson et al., 2012; Schelini et al., 2012; Holmgaard et al., 2013).

**Table 1:** One-year mortality in COPD.

Authors, year	Patients	One-year mortality	Risk factors
Connors et al. (1996)	1,016 patients with severe exacerbation	43%	FEV1, BMI, Age, PaO2
Almagro et al. (2002)	135 patients after severe exacerbation	22%	Age, women, SGRQ, previous severe exacerbations, PaCO2
Groenewegen, Schols & Wouters (2003)	171 patients with severe exacerbation	23%	PaCO2, Age
Kim, Clark & Camargo (2006)	482 patients after ED visit	23%	Age, comorbidities, previous severe exacerbations
Coleta et al. (2008)	78 patients using LTOT	15.4%	BDI, PaO2, PaCO2, SGRQ
Fan et al. (2007)	603 patients	7.5%	
Gershon et al. (2010)	>30,000 patients from Canadian register	4.3–5.7%	
Piquet et al. (2013)	1,824 patients with severe exacerbation	16.8%	Age, lower BMI, lung cancer, cardiovascular comorbidity, previous severe exacerbations, LTOT
Ho et al. (2014)	4,029 patients with severe exacerbations	22%	Age, comorbidity
Müllerova et al. (2015)	2,138 patients	5.1% in patients without severe exacerbation and 14.6% in patients with severe exacerbation	Severe exacerbations, longer hospital stay

The aim of this study is to examine the one-year mortality in COPD patients after severe exacerbation and the correlation between mortality and patients’ characteristics and comorbidities.

## Material and Methods

152 COPD patients hospitalized for COPD exacerbation were evaluated for presence of MS, DM, and hypovitaminosis D according to the following:

• MS: if at least 3 of the following were present: 1. Waist circumference > 102 cm in males, >88 cm in females; 2. Elevated triglycerides > 1.7 mmol/L (or on therapy); 3. HDL < 1.0 mmol/L in males, <1.3 mmol/L in females (or on therapy); 4. Blood pressure: systolic ≥ 130 and/or diastolic ≥ 85 mm Hg (or on therapy); 5. Fasting blood glucose > 5.5 mmol/L (or on therapy) (Alberti et al., 2009).
• DM: fasting plasma glucose ≥ 7.0 mmol/L OR 2-h plasma glucose ≥ 11.1 mmol/L during an oral glucose tolerance test (OGTT) OR HbA1c ≥ 6.5% OR on therapy (American Diabetes Association, 2012);
• Vitamin D deficiency: 25(OH)D <25 nmol/L; vitamin D insufficiency: 25(OH)D between 25 and 50 nmol/L; vitamin D sufficiency: 25(OH)D > 50 nmol/L (Borissova et al., 2012).

COPD diagnosis was established according to the Global Initiative for Chronic Obstructive Lung Disease (GOLD) criteria (GOLD, 2016). Demographic variables such as age, sex, smoking status and number of pack-years were collected. CAT and mMRC questionnaires were completed by all patients. Pre- and post-bronchodilatory spirometry was performed, according to ERS/ATS recommendations (Miller et al., 2005). Blood pressure was measured according to the American Heart Association guidelines (Pickering et al., 2005). Taking antihypertensive medications is considered as presence of arterial hypertension. The number of severe exacerbations (hospitalizations) and moderate exacerbations (worsening of pulmonary symptoms with antibiotic or/and systemic steroid treatment without hospitalization) (GOLD, 2016) in the previous year as well as the length of current hospital stay (in days) were recorded.

All patients with postbronchodilator obstruction (FEV1/FVC <  0.70) were considered eligible. The patients who did not comply with the study procedures were excluded. Informed consent was obtained by all participants.

One-year mortality was established from the national death register. Survival after hospital discharge was calculated in weeks. Statistical analyses were performed with the SPSS package for Windows software, version 22.0 (SPSS Inc., Chicago, IL, USA). Continuous variables were shown as mean ± standard deviation and 95 Confidence intervals (95%CI) and categorical variables (as percentages). The chi-squared test was used to assess the associations between categorical variables. Continuous variables were tested for normality by Shapiro–Wilk test. For normally distributed variables, independent-samples *T* test for two samples and analysis of variance (ANOVA) for more than two samples were used for determining differences between the groups. The Mann–Whitney *U* test was used for abnormally distributed variables with two samples and the Kruskal–Wallis test for variables with more than two samples. The Kaplan–Meier method was used for analyzing survival. Cox regression was used examine the importance of various covariates in the survival.

More detailed explanation of the study methods and sample characteristics, including prevalence of the comorbidities and their impact on COPD in this group of patients, are published elsewhere (Mekov et al., 2015c; Mekov et al., 2015b; Mekov et al., 2015a).

Clinical Center for Pulmonary Diseases Ethical Committee approved the study: protocol 76/25.11.2015.

## Results

### Sample characteristics

A total of 152 COPD patients admitted for COPD exacerbation were recruited from University Specialized Hospital for Active Treatment of Pulmonary Diseases ‘Saint Sofia,’ Sofia, Bulgaria. The mean age of the patients was 65 ± 10 years. There were 108 males (71.1%) and 44 females (28.9%). Mean post-bronchodilator FEV_1_ was 55.3 ± 19.5%. According to smoking patterns, the patients were: 15.8% never-smokers; 57.9% were former smokers, and 26.3% were current smokers. Mean BMI was 27.3 ± 5.6. 127 patients (83.6%) were on treatment with inhalatory corticosteroids. One-year mortality was 7.2% (11/152).

**Figure 1:**
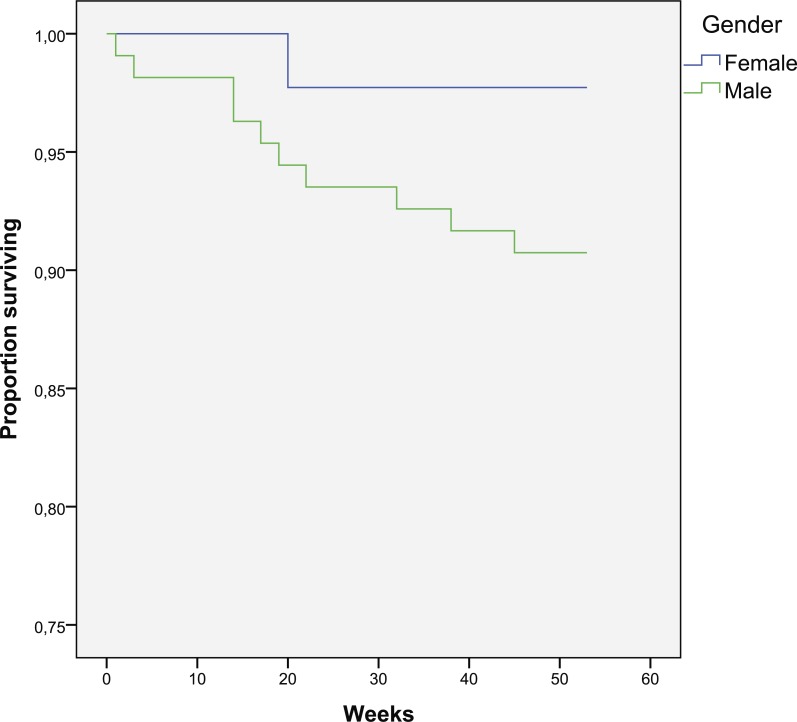
Kaplan–Meier curve for one-year survival depending on gender (in weeks).

### Mortality: role of demographic characteristics and smoking

The patients who died were older (72 vs. 65 years). Cox regression shows a 7.7% increase in mortality rate for every one year of age (*p* = 0.031). There is a trend for a slight increase in death in males (9.3 vs. 2.3%, *p* = 0.135) (Fig. 1).

Ever-smokers (both current and former) had one-year mortality of 7.0%, whereas never smokers—8.3%. Smoking is not a risk factor for increased one-year mortality, both for current and ex-smokers (*p* = 0.821).

BMI is also not a risk factor for increased mortality when tested as a scale variable and when grouped in four groups (BMI less than 18.5; 18.5–24.99; 25–29.99; and more than 30) or two groups (BMI less than 30; BMI more than 30) (all *p* < 0.10).

### Mortality: role of exacerbations and duration of hospital stay

**Figure 2:**
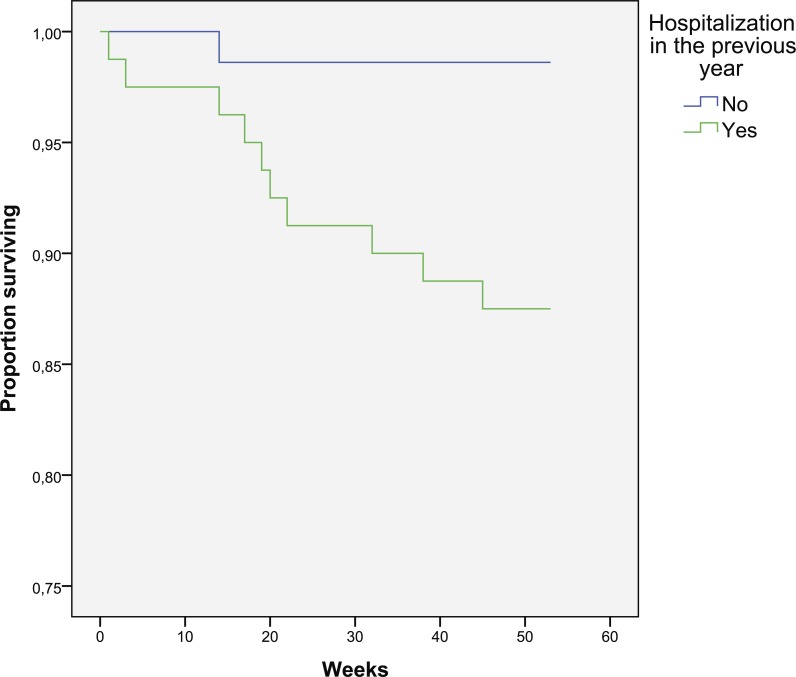
Kaplan–Meier curve for one-year survival depending on the presence of severe exacerbation in the previous year (in weeks).

The patients who died had more severe exacerbation last year (2.18 vs. 1.84). The presence of severe exacerbation in previous year is risk factor for mortality (12.5% vs. 1.4%, *p* = 0.009) (Fig. 2). The duration of hospital stay was not related to survival (*p* = 0.416).

**Figure 3:**
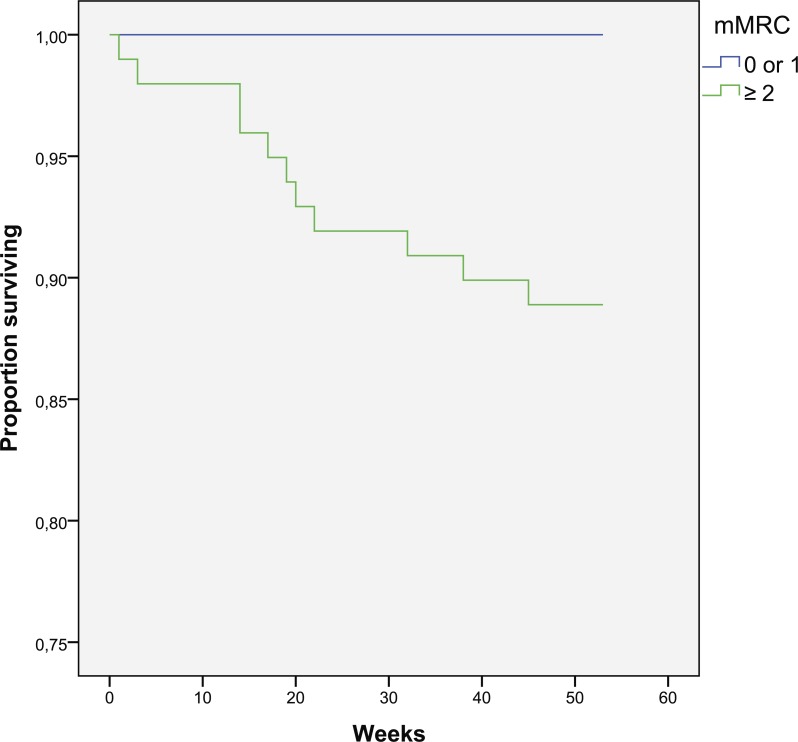
Kaplan–Meier curve for one-year survival depending on mMRC results (in weeks).

### Mortality: role of quality of life

**Figure 4:**
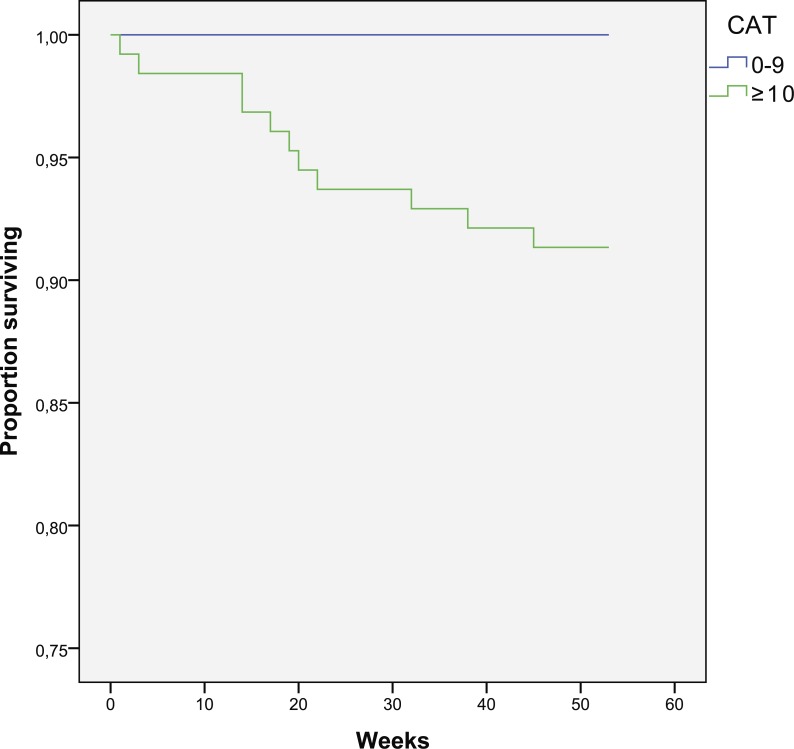
Kaplan–Meier curve for one-year survival depending on CAT results (in weeks).

There was a 0% mortality in the group with mMRC < 2 (0 vs. 11.1%, *p* = 0.013) (Fig. 3). There is a trend for increased death in the group with CAT > 10 (8.7 vs. 0%, *p* = 0.133) (Fig. 4). Cox regression shows 8.1% increase in mortality rate for every 1 CAT point increase (*p* = 0.035).

The patients who died had a worse quality of life, measured with total CAT score (22 vs. 17), CAT3 (3.64 vs. 2.57), CAT4 (4.55 vs. 3.50), CAT6 (2.91 vs. 1.48), and CAT8 (3.64 vs. 2.67) questions.

Based on CAT and mMRC results, there is a trend for increased mortality in patients with GOLD grade D, compared to grade C (8.3% vs. 0%, *p* = 0.2).

**Figure 5:**
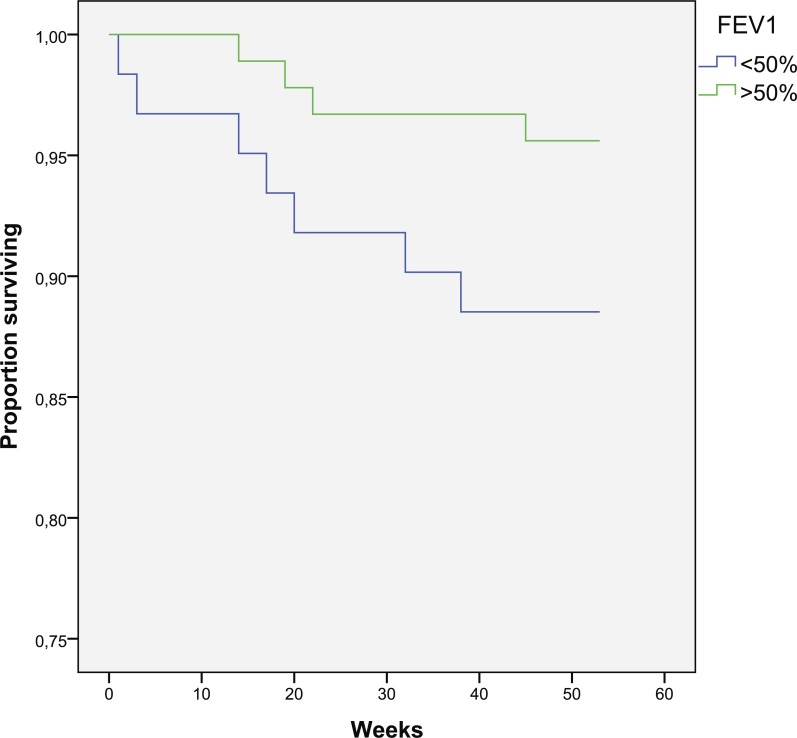
Kaplan–Meier curve for one-year survival depending on FEV1 (in weeks).

### Mortality: role of lung function

There is a trend for increased mortality in the group with FEV1 < 50% when compared to patients with FEV1 > 50% (11.5 vs. 4.4%, *p* = 0.094) (Fig. 5). Cox regression shows 3.7% increase in mortality rate for every 1% decrease in FEV1 (*p* = 0.036) and 5.2% increase in mortality rate for every 1% decrease in PEF (*p* = 0.01). FVC and FEV1/FVC are not predictors for mortality (*p* < 0.1).

**Figure 6:**
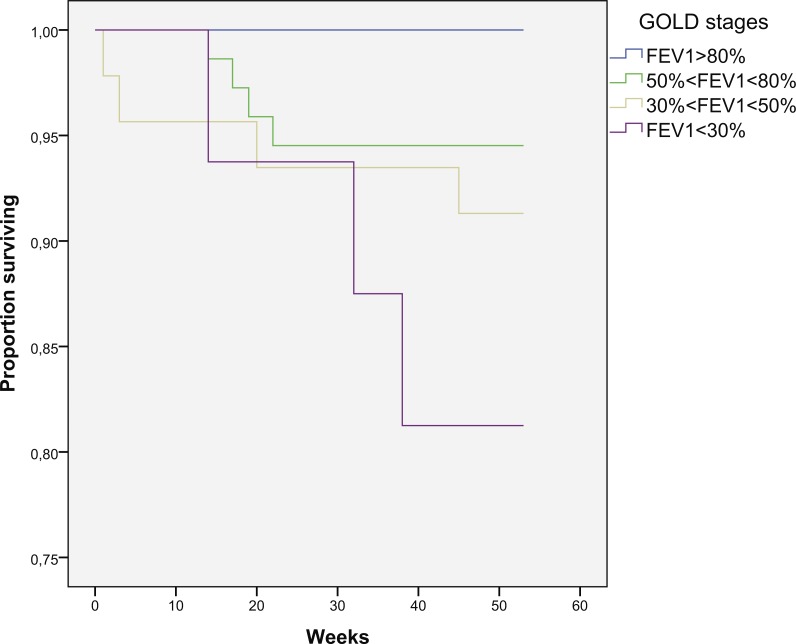
Kaplan–Meier curve for one-year survival depending on GOLD stages (in weeks).

Mortality increases with decreasing in FEV1 being 0% in patients with FEV1 > 80%, 5.5% in patients with FEV1 between 50% and 80%, 8.7% in patients with FEV1 between 30% and 50% and 18.7% in patients with FEV1 < 30%, but this difference is not statistically significant (*p* = 0.18) (Fig. 6).

The patients who died had worse lung function—FEV1 (43 vs. 56%) and PEF (37 vs. 51%).

### Mortality: role of comorbidities

DM, MS, and vitamin D status are not predictors for one-year mortality (all *p* < 0.1). However there is an increased trend for mortality in patients with arterial hypertension (9.5% vs. 2.1%, *p* = 0.107). Cox regression shows that vitamin D levels and HbA1c are not predictors for mortality. Two MS components also show dissociation trend for mortality—HDL (12.5% vs. 6.2%, *p* = 0.258) and interestingly, increased triglycerides shows protective features (2.2% vs. 9.3%, *p* = 0.124).

## Discussion

This study finds relatively low overall one-year mortality in COPD patients after surviving severe exacerbation. This could be attributed to young age of the group (mean 65 years), high duration of hospital stay (mean 7.5 days), and high FEV1 (55%). Second, this group does not include in-hospital mortality because all patients are examined before discharging at stable condition in order to assess comorbidities as accurate as possible. Nonetheless, this study supports the downward mortality trend in the recent studies which could be attributed to improved care in last decade, particularly in assessing and managing cardiovascular risk and comorbidities.

**Figure 7:**
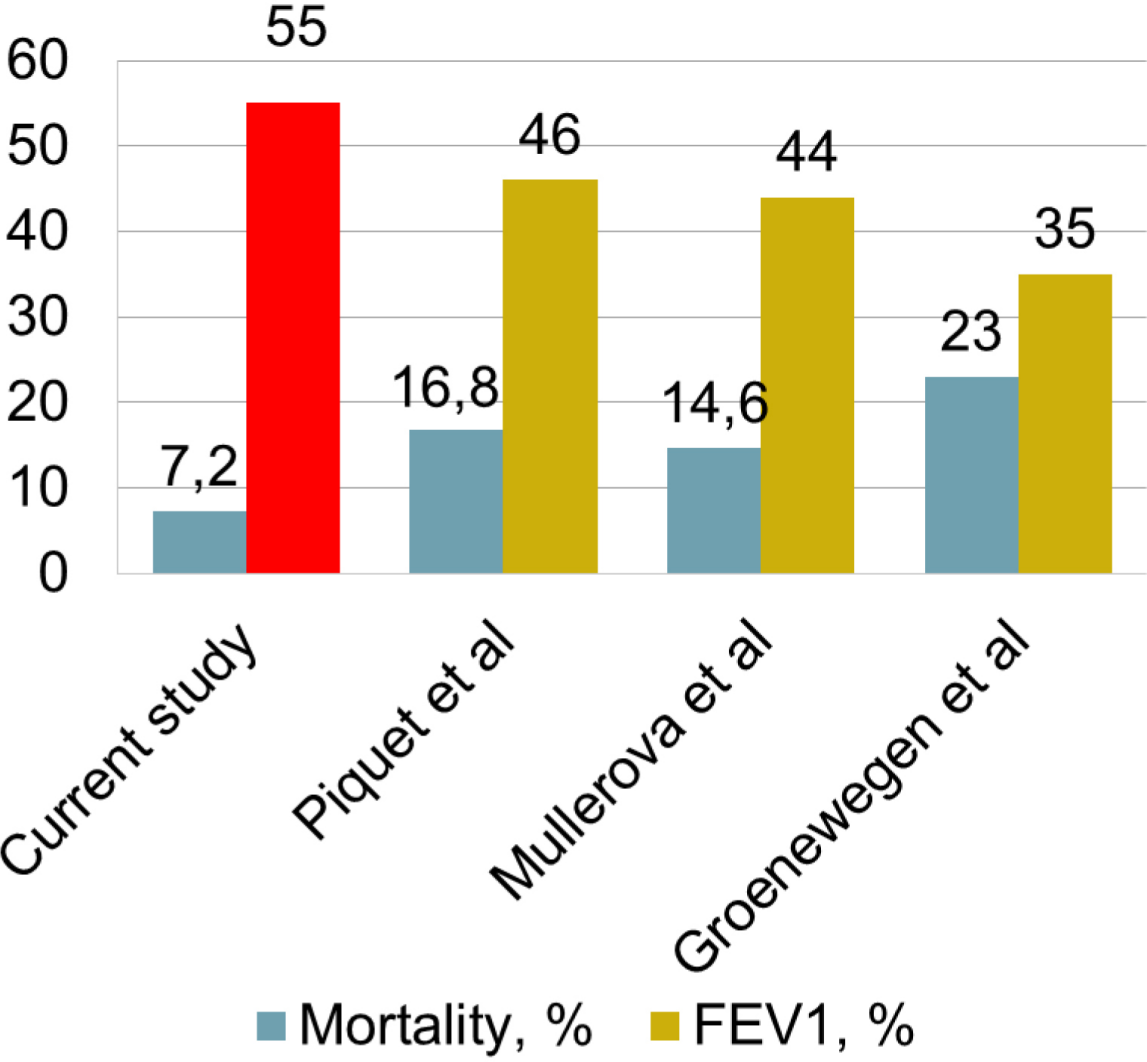
Association between mortality and FEV1. FEV1 (%) in the current study shown in red.

**Figure 8:**
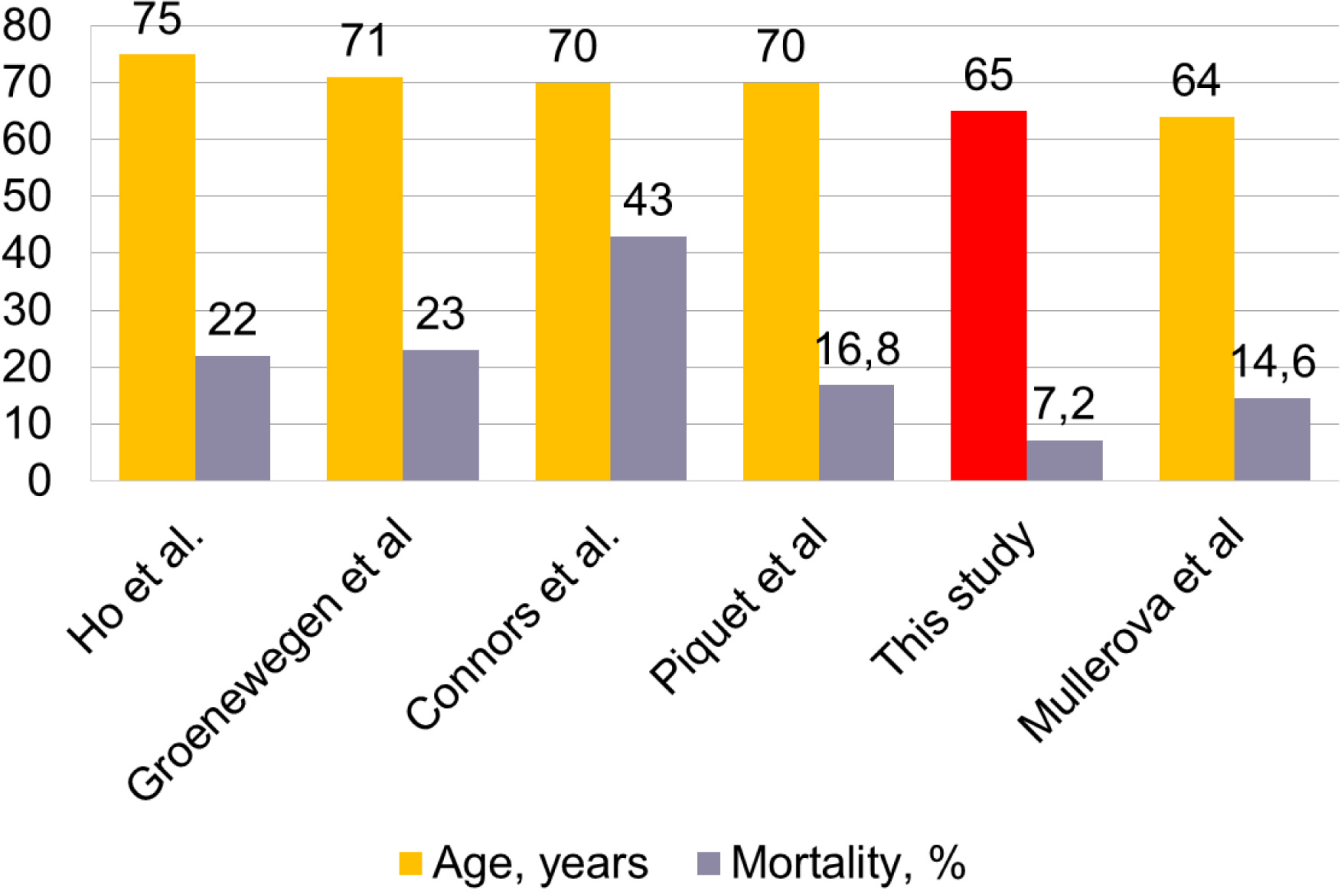
Association between mortality and Age. Age (years) in the current study shown in red.

Although many studies show severe exacerbations as a risk factor for increased mortality (Almagro et al., 2002; Kim, Clark & Camargo, 2006; Piquet et al., 2013; Müllerova et al., 2015), this study shows that sample characteristics are also important. Comparing the available data of studies mortality shows association with age and FEV1 (Figs. 7 and 8). This correlation is stronger and inverse for FEV1 and direct and less clear for age.

This study confirms age as a risk factor for one-year mortality as shown by other authors (Connors et al., 1996; Almagro et al., 2002; Groenewegen, Schols & Wouters, 2003; Kim, Clark & Camargo, 2006; Piquet et al., 2013; Ho et al., 2014). Male gender is also associated with increased mortality, although not statistically significant. Although studies find correlation between gender and mortality, some of them suggest increased risk in males (Faustini et al., 2008; Suissa, Dell’Aniello & Ernst, 2012), while others in females (Almagro et al., 2002).

The presence of severe exacerbation in the previous year is a risk factor for mortality, which confirms the results from other studies (Almagro et al., 2002; Kim, Clark & Camargo, 2006; Piquet et al., 2013; Müllerova et al., 2015). However, in this study, the number of moderate exacerbations, the total number of exacerbations, and the duration of the hospital stay were not related to mortality, as suggested by other authors (Müllerova et al., 2015).

Reduced quality of life is a risk factor for mortality which confirms previous findings (Almagro et al., 2002; Coleta et al., 2008). This study finds mMRC being more strong predictor than CAT, but there is 0% mortality in patients with less symptoms (CAT < 10) or dyspnea (mMRC < 2). The patients who died had worse quality of life, measured with total CAT score with highest impact on CAT3 (chest tightness), CAT4 (walking up), CAT6 (confidence), and CAT8 (energy) questions. There is also a trend for increased mortality in patients with GOLD grade D, compared to grade C. It is notable that grade C one-year mortality is 0% in this study.

Reduced FEV1 and PEF, but not FVC, correlate with increased mortality. Mortality increases with advancing of the disease through GOLD stages although not reaching statistical significance. It is notable that there was 0% mortality in GOLD stage 1 group (FEV1 > 80%).

Interestingly, the presence of comorbid conditions such as hypovitaminosis D, DM or MS is not associated with increased one-year mortality. This finding could be attributed to the short investigation period and low mortality rate.

Respiratory failure is significant, but not the only major death cause in COPD. Other common causes of death in these patients were cardiovascular disease and lung cancer (Sin et al., 2006), although the underlying mechanisms are not fully understood. A possible explanation could be chronic systemic and pulmonary inflammation. An interesting hypothesis on molecular level is increased reactive oxygen species formation and oxidative stress (Zuo et al., 2012). This could lead to increased TNF*α* levels, which reduce skeletal muscle force and resistance to fatigue (Zuo, Nogueira & Hogan, 2011). As a systemic inflammatory disorder, COPD is associated with respiratory muscle dysfunction (Wouters, 2002) which could be further worsened by this mechanism and contribute to the mortality. The recognition and treating of systemic COPD manifestations could be associated with the reduced mortality, showed in recent studies.

One of the important strengths of this study is the precise measurement and investigation of many outcomes, including demographic characteristics, number of exacerbations in the previous year, duration of hospital stay, lung function and quality of life. Second, it shows the influence of a common comorbidities, with focus on endocrine ones such as vitamin D, DM, and MS which are diagnosed according to the international guidelines and thus, minimizing the risk of bias.

The weak side of this study is a relatively small number of participants which may result in lack of power for some of the outcomes. However, it provides excellent information about the risk factors for one-year mortality of these patients. Nonetheless, some of the cited studies have a similar number of participants.

## Conclusions

This study finds relatively low one-year mortality in COPD patients after surviving severe exacerbation. Grade C and FEV1 >  80% may be factors for good prognosis. Risk factors for increased mortality are age, FEV1 value, severe exacerbation in previous year, and reduced quality of life.

##  Supplemental Information

10.7717/peerj.2788/supp-1 Supplemental Information 1 Raw data used for the statistical analyses Survival time in weeks (‘Survival’) was used if event was true (Death_year1 = 1). Presence of comorbidities (‘MS0_1’, ‘vitD50’, ‘diabetFINAL’ and ‘AH_th’), lung function (‘postFVC’, ‘postFEV’), number of exacerbations (‘Hosp’ for severe exacerbations, ‘AB’ for moderate exacerbations and ‘TotalEx’ for all exacerbations), quality of life (‘mMRC’, ‘CAT1-8’, ‘CATTotal’) were used for risk factor analyses. ‘Age’ and ‘sex’ as demographics are self-explanatory.

## Additional Information and Declarations

### Data Availability

The following information was supplied regarding data availability: The raw data has been supplied as Data S1.
